# Quality of life of patients with chronic lymphocytic leukaemia in the Netherlands: results of a longitudinal multicentre study

**DOI:** 10.1007/s11136-015-1039-y

**Published:** 2015-07-24

**Authors:** K. M. Holtzer-Goor, M. R. Schaafsma, P. Joosten, E. F. M. Posthuma, S. Wittebol, P. C. Huijgens, E. J. M. Mattijssen, G. Vreugdenhil, H. Visser, W. G. Peters, Z. Erjavec, P. W. Wijermans, S. M. G. J. Daenen, K. G. van der Hem, M. H. J. van Oers, C. A. Uyl-de Groot

**Affiliations:** Department of Health Policy and Management, Erasmus University Rotterdam, Institute for Medical Technology Assessment, P.O. Box 1738, 3000DR Rotterdam, The Netherlands; Medisch Spectrum Twente, Enschede, The Netherlands; St. Antonius Ziekenhuis Nieuwegein, Nieuwegein, The Netherlands; University Medical Centre Utrecht, Utrecht, The Netherlands; Medisch Centrum Leeuwarden, Leeuwarden, The Netherlands; Leiden University Medical Center, Leiden, The Netherlands; Meander Medisch Centrum, Amersfoort, The Netherlands; VU University Medical Center, Amsterdam, The Netherlands; Reinier de Graaf Groep, Delft, The Netherlands; Ziekenhuis Rijnstate, Arnhem, The Netherlands; MÃ¡xima Medisch Centrum, Veldhoven, The Netherlands; Medisch Centrum Alkmaar, Alkmaar, The Netherlands; Catharina-ziekenhuis, Eindhoven, The Netherlands; Ommelander Ziekenhuis Groep, Delfzijl, The Netherlands; Hagaziekenhuis, Den Haag, The Netherlands; UMCG, University of Groningen, Groningen, The Netherlands; Zaans Medisch Centrum, Zaandam, The Netherlands; Academic Medical Center, University of Amsterdam, Amsterdam, The Netherlands; MUMC+, Maastricht, The Netherlands

**Keywords:** Leukaemia, Lymphocytic, Chronic, B cell, Quality of life, Area under curve, Observational study

## Abstract

**Purpose:**

To describe the health-related quality of life (HRQoL) of an unselected population of patients with chronic lymphocytic leukaemia (CLL) including untreated patients.

**Methods:**

HRQoL was measured by the EORTC QLQ-C30 including the CLL16 module, EQ-5D, and VAS in an observational study over multiple years. All HRQoL measurements per patient were connected and analysed using area under the curve analysis over the entire study duration. The total patient group was compared with the general population, and three groups of CLL patients were described separately, i.e. patients without any active treatment (“watch and wait”), chlorambucil treatment only, and patients with other treatment(s).

**Results:**

HRQoL in the total group of CLL patients was compromised when compared with age- and gender-matched norm scores of the general population. CLL patients scored statistically worse on the VAS and utility score of the EQ-5D, all functioning scales of the EORTC QLQ-C30, and the symptoms of fatigue, dyspnoea, sleeping disturbance, appetite loss, and financial difficulties. In untreated patients, the HRQoL was slightly reduced. In all treatment stages, HRQoL was compromised considerably. Patients treated with chlorambucil only scored worse on the EORTC QLQ-C30 than patients who were treated with other treatments with regard to emotional functioning, cognitive functioning, bruises, uncomfortable stomach, and apathy.

**Conclusions:**

CLL patients differ most from the general population on role functioning, fatigue, concerns about future health, and having not enough energy. Once treatment is indicated, HRQoL becomes considerably compromised. This applies to all treatments, including chlorambucil, which is considered to be a mild treatment.

**Electronic supplementary material:**

The online version of this article (doi:10.1007/s11136-015-1039-y) contains supplementary material, which is available to authorized users.

## Introduction

Chronic lymphocytic leukaemia (CLL) is the most common type of leukaemia occurring in the Western world, affecting around 3–6 people per 100,000 persons [1–3]. Early symptoms of CLL are minimal and diagnosis often follows the incidental finding of a high lymphocyte blood count or lymph node swelling. Unlike most types of cancer, the majority of CLL patients will not be treated immediately after diagnosis but will be monitored through a “watch and wait” approach [4]. Only upon disease progression and/or the development of CLL-related symptoms such as fatigue, weight loss, malaise, bleeding, and recurrent or persistent infections [5, 6], treatment is indicated.

Disease-related symptoms, toxic effects of therapy, and the awareness of living with an incurable disease [7] can have a profound impact on health-related quality of life (HRQoL). Despite these effects, little is known about the HRQoL of patients living with CLL [7–9]. Currently, nearly all available information is obtained during clinical trials which also studied the influence of treatment with chemotherapy on HRQoL [10–13]. However, the generalisability of these studies is limited because these studies only enrol patients in need of treatment. In addition, they use strict inclusion and strict exclusion criteria, e.g. often excluding patients over the age of 65.

The measurement of HRQoL in clinical trials which enrol mostly younger patients in need of treatment is valuable for comparison of treatments with regard to their effect on HRQoL and the course of these effects over time, i.e. from the start of treatment till the start of next treatment. From the available studies, we know that the HRQoL of patients during and after treatment with fludarabine plus cyclophosphamide (FC) does not differ from that of patients treated with fludarabine monotherapy on global health score, physical and emotional functioning, and fatigue. Patients treated with FC score worse on nausea and vomiting during treatment, and better (but not significantly better) after treatment than patients treated with fludarabine monotherapy [11, 12]. However, these clinical trials do not allow a conclusion with regard to the HRQoL in patients who are not in need of treatment yet.

That information would be valuable since one-third of all CLL patients [14] will not progress to treatment even over decades. Current study provides an indication of the type of symptoms that treatment-naïve patients experience and the limitations in daily functioning that occur. The comparison of the HRQoL in untreated patients versus those who just started treatment might give some indication of the impact of starting first-line treatment on HRQoL. When the HRQoL of untreated patients is already severely compromised, the impact of expected side effects during treatment on HRQoL is not likely to have a decisive role in the decision whether to start treatment. In the opposite situation, the expected impact of starting treatment on HRQoL should be seriously considered in the decision whether to start treatment or not.

None of the available studies that address HRQoL in the whole CLL population [15–17] measured the HRQoL over a period longer than 1 year. In order to fill this gap, we conducted a longitudinal, multicentre observational study including a HRQoL study.

## Patients and methods

### Inclusion and exclusion criteria

Nineteen hospitals in the Netherlands invited patients with CLL for participation in an observational study addressing the management of CLL, costs, and HRQoL [18]. Patients aged 18 years or older diagnosed with CLL could enter the study if he or she did not suffer from another serious malignant disease or previous malignancy, had a complete record, and gave informed consent. Patients who developed a non-CLL-related malignancy were censored at the time of its diagnosis.

### Quality of life

Patients who participated in the HRQoL study received a HRQoL questionnaire at the start, halfway through, and at the end of therapy from their treating specialists. Additional questionnaires were sent every 6 months in the periods without treatment to get information about the HRQoL in the period before treatment and between treatments. Since chlorambucil was frequently administered continuously for a long and not predetermined period of time, we choose to send questionnaires during this treatment every 6 months as well, to get more information about the HRQoL during the whole period of treatment.

The instruments employed in the HRQoL assessment were the European Organisation for Research and Treatment of Cancer (EORTC) QLQ-C30 accompanying CLL-specific module [19] and a modified version of the EQ-5D in which five response levels replaced the original three levels [20] as suggested and investigated by Kind and Macran [21].

### EORTC QLQ-C30

The EORTC QLQ-C30 has been developed by the EORTC Quality of Life Study Group to assess the QoL of patients with cancer [19]. The core instrument includes 30 questions covering many QoL issues related to cancer patients in general and can be supplemented by a diagnosis-specific module [22].

The questionnaire EORTC QLQ-C30 incorporates five functional scales, three symptom scales, a global quality of life scale (two items), and six single items. The functional scales are physical functioning, role functioning, emotional functioning, cognitive functioning, and social functioning. The symptom scales are fatigue, nausea and vomiting, and pain. Dyspnoea (shortness of breath), sleeping (disturbance), appetite loss, constipation, diarrhoea, and financial difficulties are the six single items. According to the EORTC scoring manual, scores were linearly transformed to a 0–100 scale [23]. A higher score on the functional scales and global quality of life scales meant better functioning and quality of life, whereas a higher score on the symptom scales meant more complaints. Differences in scale scores of 10 points or more were considered clinically meaningful [24].

In this study, the core questionnaire EORTC QLQ-C30 was supplemented by the CLL-specific module [25]. The module is used to describe aspects of CLL that are not included in the core questionnaire and provides information about several domains. There are three multi-item scales, i.e. fatigue, treatment side effects and disease symptoms, infections, and two single item scales on social activities and future health worries. However the module is not yet officially published, the score on the scales cannot be calculated [25], and the average score—ranging from 1 (not at all) to 4 (very much)—on the items can be described.

### Modified version of the EQ-5D

The EQ-5D measures the general HRQoL and is therefore not influenced by CLL only. At the time of start of the study, a five-level EQ-5D had been developed since the original three-level EQ-5D was not sensitive enough for smaller changes in HRQoL. Since patients with CLL in general experience a high level of HRQoL [15], at least until they reach the advanced stages, it was hypothesised that this expanded five-level classification might provide a more sensitive measure of change in health status than the original three-level EQ-5D (EQ-5D_3_).

The modified version of the EQ-5D (EQ-5D_5_) [21] comprised the same two items as the EQ-5D_3_: a visual analogue scale (VAS) providing a single overall summary score of HRQoL and descriptive classification with five dimensions (mobility, self-care, usual activities, pain/discomfort, anxiety/depression). However, the descriptive classification of the EQ-5D_5_ contained five levels, rather than the standard three levels. The two additional levels were unlabelled [21]. It can be seen as the predecessor of the labelled five-level version of the EQ-5D [26], which did not exist at the start of our study yet.

The responses on the descriptive classification can be translated to a utility score, which is a value that reflects an individual’s preference for a certain health outcome with zero reflecting states of health equivalent to death and one reflecting perfect health. Utility values for the EQ-5D_5_ states have never been determined, as this instrument has been replaced by a five-level labelled version. We calculated utility values following the suggestion of the creator of the EQ-5D_5_ [21]. The known utility values for the levels 1, 2, and 3 of the EQ-5D_3_ were used for the levels 1, 3, and 5 of the EQ-5D_5_, and the additional two levels were generated assuming the midpoint value between the standard two tariff values using an adaptation of the Dutch three-level tariff [27].

### Statistical analysis

The HRQoL of a CLL patient over time was calculated by connecting all measurements per patient using area under the curve analysis over the entire study duration. To enable the comparison of patients, we presented area under the curve values corrected for the follow-up duration per patient. For each patient, an individual norm score was derived from age- and gender-matched scores of the general population on the EQ-5D [28] and EORTC QLQ-C30 [29]. These two studies, as reported in Refs. [28] and [29], used a panel consisting of more than 2000 Dutch households, representative of the Dutch-speaking non-institutionalised population in the Netherlands.

Patient scores were compared with norm scores using *t* test or nonparametric test for related samples (significant when *p* < 0.05). Patient scores of three patient groups (patients without any active treatment, patients treated with chlorambucil only, and other patients) were compared using the Kruskal–Wallis test.

Subsequently, we chose to focus on the HRQoL during two treatment phases. First, we focused on the questionnaires completed during the watch and wait phase since data on this subject are scarce, and second on the questionnaires filled in during chlorambucil treatment because this was the most frequently administered treatment in our study. The results of both phases were described in a separate section and compared using Kruskal–Wallis test or *t* test depending on the variable distribution.

## Results

### Patient characteristics

Informed consent for participation was given by 173 CLL patients. Of these, 13 patients (6 %) were excluded from the analysis for the following reasons: eight patients did not meet the inclusion criteria after all; one patient chart was missing; and one patient withdrew himself from the study. Additionally, one hospital dropped out of the study, leaving three patients with incomplete follow-up data.

Of the 160 evaluable patients, 144 patients (90 %) participated in the HRQoL study. Table 1 presents patient characteristics of these 144 patients as a whole and per patient group: patients who did not receive any active treatment during the study period, patients who only received chlorambucil, and patients with other or more treatments. It also presents the characteristics of the patients who did not participate in the HRQoL part of the study.

**Table 1:** Patient characteristics

	All participants (*n* = 144)	Patients without any active treatment (*n* = 59)	Patients treated with CLB only (*n* = 28)	Other patients (*n* = 57)	Non-participants in HRQoL study (*n* = 16)
Age at diagnosis					
Mean (SD)	62.6 (10.5)	64.1 (9.3)	63.6 (12.1)	60.5 (10.6)	71.0 (8.6)
Median	63	64	66	61	69
Range	30–86	34–82	30–86	38–85	56–84
Gender (% male)	62.5	59.3	50.0	71.9	62.5
Patients (%) with first- or second-degree relatives with leukaemia or lymphoma	9.0	6.8	10.7	10.5	0.0
Binet Stage (%)					
A	70.8	94.9	67.9	47.4	81.2
A progressive	2.1	0	0	5.3	6.3
B	16.0	1.7	21.4	28.1	12.5
C	11.1	3.4	10.7	19.3	
B-symptoms (yes %)	12.5	5.1	10.7	21.1	13.3
Involvement of spleen (yes %)	27.8	10.2	42.9	38.6	26.7
Comorbidities (yes %)	27.8	20.3	39.3	29.8	43.7
WHO performance score (%)					
0	78.5	84.7	71.4	75.4	75.0
1	19.4	15.3	25.0	21.1	18.8
2	0	0	0	0	6.3
n.a.	2.1	0	3.6	3.5	0

*n.a.* not available, *CLB* chlorambucil, *HRQoL* health-related quality of life

The mean age at diagnosis of all patients was 62.6 years (SD = 10.5) of whom the majority were male (63 %). On average, male patients were younger at diagnosis (60.8 years, SD = 10.1) than female patients (65.5 year, SD = 10.5). Age at diagnosis did not differ significantly between the patient groups.

From diagnosis until the end of the HRQoL study, 85 patients received active treatment (59 %). Seventy-three patients started treatment before the start of the HRQoL study and 12 patients started their first-line treatment during the study period. Eighty-five per cent of all patients who received active treatment, were treated initially with chlorambucil with or without prednisone. Other initial treatments were chlorambucil–vincristine–prednisone (CVP) (7 %), fludarabine (2 %), fludarabine–cyclophosphamide (FC, 2 %), rituximab plus CVP (R-CVP, 1 %), cyclophosphamide (1 %), and cyclophosphamide–doxorubicin–teniposide–prednisone with bleomycin–vincristine + radiotherapy (1 %). Fifty-three patients also received subsequent line(s) of treatment. Second line treatment was fludarabine monotherapy in most patients (23 patients, 43 %). Other second line treatments were: CVP (17 %), FC (8 %), FCR (8 %), R-CVP (8 %), chlorambucil plus prednisone (6 %), rituximab (4 %), R-CHOP (4 %), CVPP (2 %) and fludarabine–rituximab (FR, 2 %).

**Fig. 1:**
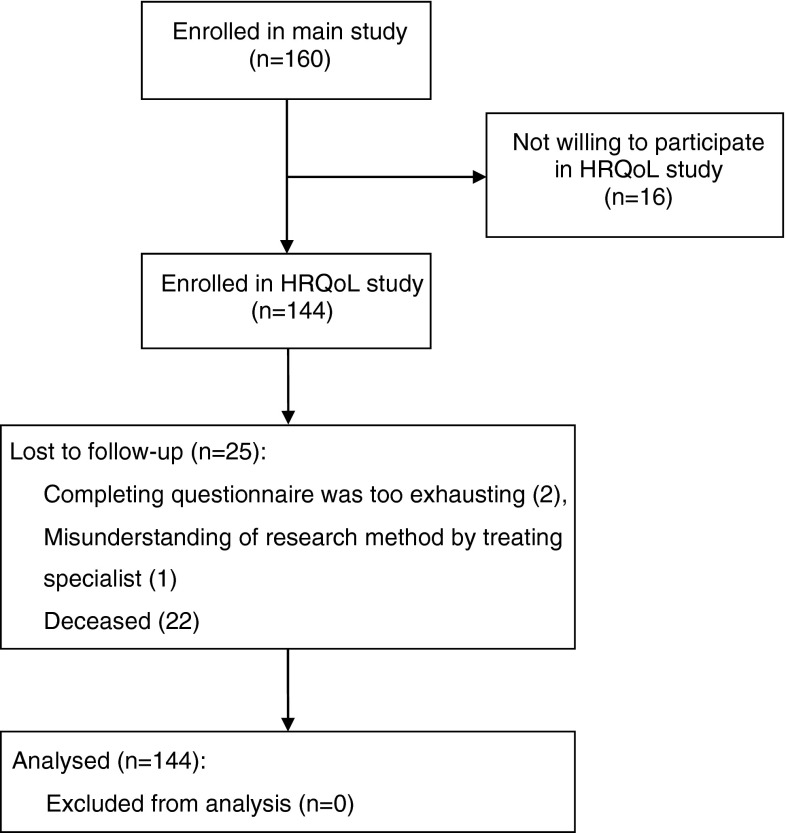
Patient flow chart

Patients were diagnosed for on average 3.9 years at the time of their first questionnaire. Their last questionnaire was on average completed 2.6 years later, at 6.5 years since diagnosis. The mean number of questionnaires was 5.7 per patient, and 127 patients (88.2 %) completed three or more questionnaires. For 25 patients, we did not have information during the complete follow-up duration of the study (see Fig. 1).

### Quality of life during total study

Table 2 summarises the results on all instruments used for the total CLL population and for the three patient groups that were described before.

**Table 2:** Average patient* and norm scores on EQ-5D_5_, EORTC QLQ-C30 and EORTC QLQ-CLL16 (SD) of the total CLL population, and the three patient groups

	Total group of CLL patients		Patient groups		
	Total group (*n* = 144)	Age- and gender-matched norm score	Patients without any active treatment (*n* = 59)	Patients with (watch and wait+) chlorambucil only (*n* = 28)	Other patients (*n* = 57)
EQ-5D_5_					
Utility	0.85 (0.1)†	0.89 (0.0)	0.89 (0.1)	0.82 (0.2)*	0.85 (0.1)†
VAS	73.5 (12.9)‡	83.1 (3.7)	77.6 (12.8)†	71.3 (12.0)‡	70.3 (12.4)‡
EORTC QLQ-C30—functioning scales					
Physical functioning	79.15 (18.1)‡	87.18 (5.9)	83.95 (16.2)	75.89 (22.3)*	75.79 (17.8)‡
Role functioning	75.44 (22.9)‡	86.57 (4.2)	81.99 (20.9)	71.30 (23.9)†	70.68 (24.8)‡
Emotional functioning	85.31 (15.3)‡	89.89 (2.0)	87.29 (13.4)	77.52 (18.3)†	87.09 (16.3)
Cognitive functioning	84.98 (16.1)‡	90.81 (2.9)	85.59 (16.3)*	76.50 (18.2)‡	88.53 (16.6)
Social functioning	85.61 (18.3)‡	93.44 (2.4)	90.60 (14.5)	82.76 (22.0)*	81.85 (21.5)‡
Global health	75.36 (13.8)	77.06 (2.7)	78.68 (13.1)	73.86 (14.7)	72.66 (14.8)*
EORTC QLQ-C30—symptoms					
Fatigue	31. 17 (21.0)‡	17.51 (3.8)	24.96 (21.4)†	36.48 (21.1)‡	34.97 (20.6)‡
Nausea vomiting	3.77 (7.7)	2.50 (1.8)	2.31 (5.0)	5.96 (9.9)	4.20 (9.3)
Pain	15.06 (17.9)	17.26 (5.6)	14.48 (18.2)	19.58 (23.0)	13.45 (15.6)
Dyspnoea	18.15 (21.7)‡	9.30 (3.1)	12.12 (17.9)	19.02 (21.4)*	23.96 (23.0)‡
Sleeping	22.07 (23.6)‡	15.18 (4.9)	20.86 (25.0)	28.85 (20.9)†	20.00 (25.4)
Appetite loss	8.36 (15.8)‡	3.48 (1.7)	3.94 (9.6)	16.92 (24.9)*	8.73 (13.9)†
Constipation	4.77 (10.5)	5.98 (2.9)	4.41 (9.6)	4.87 (9.9)	5.09 (12.3)
Diarrhoea	4.75 (9.8)	3.96 (0.9)	4.52 (11.2)	5.76 (11.3)	4.50 (7.1)
Financial difficulties	5.78 (13.8)*	3.33 (1.35)	4.77 (11.5)	5.38 (16.5)	7.03 (20.1)
EORTC QLQ-CLL16					
Weight loss	1.15 (0.5)	n.a.	1.06 (0.4)	1.34 (0.6)	1.15 (0.6)
Dry mouth	1.38 (0.8)	n.a.	1.35 (0.7)	1.61 (0.9)	1.31 (0.7)
Bruises	1.06 (0.5)	n.a.	1.05 (0.4)	1.24 (0.7)	0.98 (0.4)
Uncomfortable stomach	1.27 (0.7)	n.a.	1.24 (0.6)	1.49 (0.7)	1.20 (0.6)
Changes in temperature	1.14 (0.6)	n.a.	1.03 (0.4)	1.30 (0.7)	1.17 (0.7)
Night sweats	1.55 (0.9)	n.a.	1.42 (0.8)	1.76 (0.9)	1.58 (0.9)
Feeling sick or unwell	0.78 (0.5)	n.a.	0.68 (0.4)	0.99 (0.7)	0.79 (0.5)
Feeling apathetic	1.41 (0.7)	n.a.	1.30 (0.7)	1.71 (0.7)	1.37 (0.7)
Not enough energy	1.49 (0.8)	n.a.	1.36 (0.7)	1.79 (0.8)	1.47 (0.8)
Planning activities	1.45 (0.8)	n.a.	1.29 (0.7)	1.73 (0.9)	1.46 (0.8)
Future health concern	1.62 (0.8)	n.a.	1.50 (0.8)	1.93 (1.0)	1.59 (0.9)
Respiratory infection	1.42 (0.8)	n.a.	1.26 (0.5)	1.42 (0.7)	1.58 (0.9)
Other infection	1.26 (0.6)	n.a.	1.19 (0.6)	1.25 (0.6)	1.33 (0.8)
Repeated use antibiotics	1.26 (0.7)	n.a.	1.10 (0.5)	1.17 (0.5)	1.48 (0.9)
Worries for infection risk	1.32 (0.7)	n.a.	1.10 (0.5)	1.47 (0.8)	1.48 (0.8)

Patient scores were based on an area under the curve analysis

* *p* < 0.05, † *p* < 0.01, ‡ *p* < 0.001 for comparisons with age- and gender-matched norm scores

Taking into account the total group of CLL patients, the score on both the EQ-5D and the VAS was lower than the norm score corrected for age and gender [28]. This also applies for the subgroups of patients treated with chlorambucil only or with more/other treatments than chlorambucil. Patients who received no active treatment at all, scored lower on the VAS than the general population, but not on the utility score of the EQ-5D_5_.

The patients’ mean score and the mean norm scores per EORTC QLQ-C30 item/scale are also shown in Table 2. It identifies the significant differences of *p* < 0.05 from the norm score. Statistically significant differences are, however, not always clinically meaningful. Meaningful differences (of more than 10 points [24]) between the norm score and patients’ score were observed for role functioning and fatigue in the total group of CLL patients. This was also applicable to the subgroups of patients treated with chlorambucil only or with more/other treatments than chlorambucil. Other differences were observed for emotional and cognitive functioning, appetite loss, and sleeping in patients who only received chlorambucil, for physical and social functioning, and for dyspnoea in patients who received more or other treatments than chlorambucil. None of the significant differences for patients who did not receive any active treatment were clinically meaningful.

When looking at the total population of CLL patients that reported “a little”, “quite a bit”, or “very much” problems on the EORTC QLQ-CLL16 questionnaire, most patients reported problems on future health concern (62 % of the questionnaires), feeling to have not enough energy (50 %), and having night sweats (48 %). For all patient groups, most problems were reported on future health concern and night sweats. The subgroup of patients who were treated with more or different therapies than chlorambucil also reported many problems with respiratory infections and worries about getting infections. The subgroup with patients who only received chlorambucil had the highest (worst) total mean score over all items.

**Fig. 2:**
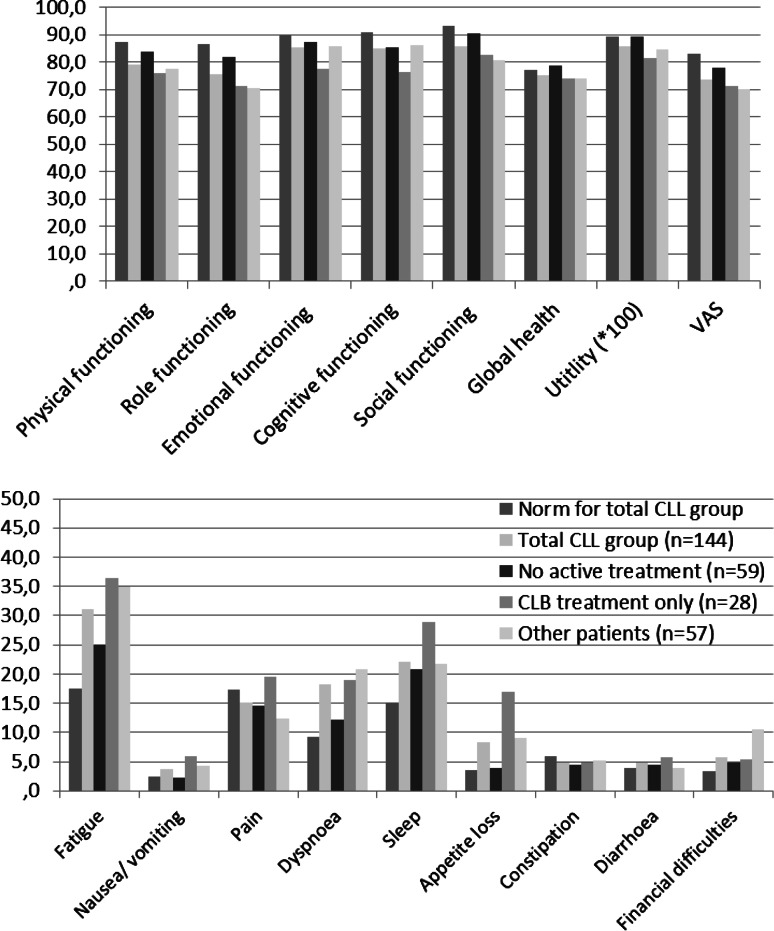
Norm scores and patient scores on the EORTC QLQ-C30 and EQ-5D_5_. Patient scores on the EORTC QLQ-C30 and EQ-5D_5_ are reported for the general population (norm score) [28, 29], the total CLL group and for the three patient groups separately. The norm scores present the mean norm score of all CLL patients in our study. *Upper figure* results of the functioning scales of the EORTC QLQ-C30 and the EQ-5D. The higher the score, the higher the quality of life (range 0–100). *Lower figure* results of the symptom scales of the EORTC QLQ-C30 The higher the score, the lower the quality of life (range 0–100). *CLB* chlorambucil

Figure 2 shows that on almost all single items and scales, the group without any active treatment (watch and wait approach only) scored best of all patient groups. Patients who were treated with chlorambucil only scored worse on HRQoL than patients who were treated with more or different treatments with regard to emotional functioning, cognitive functioning, bruises, uncomfortable stomach, and apathy (data not shown).

Being currently treated or not did influence the HRQoL. The 41 patients who filled in questionnaires during treatment and before/after treatment had a significantly lower utility score during treatment (data not shown). This pattern was also observed in the total study sample as presented elsewhere when the data were analysed per treatment line [30].

In the total population of CLL patients, scores on the VAS and EQ-5D_5_ differed significantly between the categories of WHO performance status and the presence/absence of comorbidities (see Supplemental Table 1).

### Quality of life during watch and wait phase and during treatment with chlorambucil versus general population

HRQoL results during the watch and wait phase are based on all questionnaires completed before the start of active treatment. This covers not only patients who did not receive any active treatment at all, but also the patients who received a treatment after being in the watch and wait phase. During the watch and wait phase, HRQoL can be compromised due to the illness and its related insecurity as well as by other causes like comorbidities or life events.

The HRQoL during treatment with chlorambucil covers only those questionnaires which were filled in during active treatment with chlorambucil.

Both patients in the watch and wait phase and those during treatment with chlorambucil scored lower on the VAS than the general population corrected for age and gender distribution (Table 3). The difference in utility was only significant for patients treated with chlorambucil (0.81 vs. 0.90).

**Table 3:** Average patient* and norm scores on EQ-5D_5_ and EORTC QLQ-C30 (SD) during watch and wait phase and during treatment with chlorambucil

	During watch and wait phase (*n* = 71)	Norm score watch and wait phase	*p* value***	During treatment with CLB (*n* = 42)	Norm score during treatment with CLB	*p* value***
Utility	0.88 (0.1)	0.89 (0.0)	0. 052	0.81 (0.2)**	0.90 (0.0)	*0.003*
VAS	77.4 (12.4)	82.8 (3.9)	*0.000*	69.1 (14.5)**	82.7 (3.7)	*0.000*
Physical functioning	83.2 (15.9)	86.8 (6.1)	*0.030*	79.0 (18.2)	86.2 (5.3)	*0.011*
Role functioning	79.8 (21.2)	86.3 (4.4)	*0.009*	73.3 (22.5)	85.5 (3.5)	*0.001*
Emotional functioning	86.6 (13.7)	89.7 (2.1)	0.055	78.0 (18.0)**	89.7 (2.1)	*0.000*
Cognitive functioning	85.2 (16.9)	90.9 (2.9)	*0.038*	82.6 (19.3)	90.2 (3.0)	*0.011*
Social functioning	89.9 (15.0)	93.3 (2.5)	0.051	83.5 (20.0)*	92.7 (2.2)	*0.004*
Global health	78.0 (13.6)	76.9 (2.8)	0.474	72.9 (15.4)	76.4 (2.1)	0.147
Fatigue	25.5 (20.5)	17.7 (4.0)	*0.002*	22.99 (17.8)*	18.6 (3.1)	*0.000*
Nausea vomiting	2.9 (5.9)	2.7 (1.9)	0.766	4.49 (15.6)	2.6 (1.8)	0.435
Pain	15.5 (17.6)	17.8 (5.9)	0.254	15.38 (18.3)	18.4 (4.9)	0.279
Dyspnoea	12.1 (18.6)	9.4 (3.2)	0.225	24.28 (26.1)**	9.6 (3.0)	*0.001*
Sleeping	21.6 (24.6)	15.6 (5.1)	*0.032*	26.75 (26.3)	16.1 (4.7)	*0.012*
Appetite loss	5.5 (12.4)	3.6 (1.7)	0.191	9.98 (21.9)	3.7 (1.6)	0.067
Constipation	4.3 (9.4)	6.2 (3.0)	0.085	3.48 (8.6)	6.5 (2.7)	*0.025*
Diarrhoea	4.4 (10.3)	3.9 (1.0)	0.720	3.62 (8.6)	4.2 (0.8)	0.686
Financial difficulties	5.6 (14.0)	3.4 (1.4)	0.195	5.00 (16.7)	3.5 (1.5)	0.561
Weight loss	1.2 (0.3)	n.a.		1.48 (0.3)*	n.a.	
Dry mouth	1.5 (0.7)	n.a.		1.70 (0.7)	n.a.	
Bruises	1.1 (0.3)	n.a.		1.20 (0.3)	n.a.	
Uncomfortable stomach	1.4 (0.5)	n.a.		1.53 (0.6)	n.a.	
Changes in temperature	1.1 (0.3)	n.a.		1.43 (0.3)**	n.a.	
Night sweats	1.7 (0.7)	n.a.		1.95 (0.7)	n.a.	
Feeling sick or unwell	1.3 (0.4)	n.a.		1.45 (0.4)	n.a.	
Feeling apathetic	1.5 (0.5)	n.a.		1.79 (0.6)**	n.a.	
Not enough energy	1.5 (0.6)	n.a.		1.88 (0.6)*	n.a.	
Planning activities	1.4 (0.6)	n.a.		1.74 (0.6)	n.a.	
Future health concern	1.7 (0.7)	n.a.		2.10 (0.7)	n.a.	
Respiratory infection	1.4 (0.5)	n.a.		1.78 (0.5)*	n.a.	
Other infection	1.3 (0.5)	n.a.		1.38 (0.5)	n.a.	
Repeated use antibiotics	1.3 (0.5)	n.a.		1.40 (0.5)	n.a.	
Worries for infection risk	1.3 (0.4)	n.a.		1.78 (0.4)**	n.a.	

Patient scores were based on an area under the curve analysis

* A significant difference between the watch and wait phase and treatment with chlorambucil (*p* value <0.05)

** A significant difference between the watch and wait phase and treatment with chlorambucil (*p* value <0.01)

*** A value in italics indicates a significant difference between the patient score and norm score (*p* value < 0.05)

Supplemental Table 1 shows that scores on the EQ-5D_5_ were significantly different between the categories of gender, age at diagnosis, WHO performance status, and the presence/absence of comorbidities for patients during the watch and wait phase. During treatment with chlorambucil, none of the collected patient characteristics influenced the score on the EQ-5D and VAS significantly (e.g. males vs. females).

When comparing the individual patient scores on the EORTC QLQ-C30 with the individual age and gender-adjusted norms scores, the patients’ scores were meaningfully different from the norm score on emotional and role functioning and on sleeping and dyspnoea during treatment with chlorambucil. Differences were also found for physical, cognitive, and social functioning and sleeping scales, but although statistically significant, they were not clinically meaningful. In the watch and wait phase, differences from the norm score for cognitive, role and physical functioning, fatigue, and sleeping were statistically significant, but not clinically meaningful.

With regard to the items of EORTC QLQ-CLL16 module, patients in the watch and wait phase suffered most from worries about their future health (55 % of the questionnaires), night sweats (44 %), and having not enough energy (40 %). Patients during treatment with chlorambucil suffered most from worries about their future health (78 % of the questionnaires), having not enough energy (61 %), and infection risk (56 %).

### Quality of life during watch and wait phase versus during treatment with chlorambucil

Patient characteristics of the patients who completed questionnaires during the watch and wait phase were comparable with those of the patients who completed questionnaires during treatment with chlorambucil. Age at diagnosis was 68.7 versus 67.2 years (*p* = 0.426), WHO performance status was 0 in 83.1 versus 82.9 % of the patients (*p* = 0.981), and co-morbidity was present in 26.8 versus 37.2 % of the patients (*p* = 0.195), respectively.

The HRQoL was significantly worse during treatment with chlorambucil than during the watch and wait phase for the following outcomes: utility, VAS, emotional functioning, social functioning, fatigue, dyspnoea, losing weight, changes in temperature, feeling apathetic, lack of energy, respiratory infections, and risk of infections.

Norm scores were available for the EQ-5D [28] and the EORTC QLQ-C30 [29]. The mean difference between the patients’ score and the norm score for that patient was significantly higher during treatment with chlorambucil than during the watch and wait phase for the following scales and items: emotional functioning (*p* = 0.004), fatigue (*p* = 0.021), dyspnoea (*p* = 0.003), VAS (*p* = 0.002), and utility (*p* = 0.004).

## Discussion

This longitudinal observational study showed that the HRQoL in CLL patients is compromised when compared with age- and gender-matched norm scores of the general population. Patients with CLL differed from the general population on the VAS and utility score of the EQ-5D_5_, all functioning scales of the EORTC QLQ-C30, and the symptoms of fatigue, dyspnoea, sleeping, appetite loss, and financial difficulties.

The HRQoL in untreated CLL patients is already compromised with regard to physical, role and cognitive functioning, VAS score, fatigue, and sleeping. During treatment with the most frequently administered therapy in our study (chlorambucil), patients also had dyspnoea and constipation and were compromised in their emotional and social functioning. Although we are aware that treatment is initiated only when there is a treatment indication and clinical benefits are to be expected, we conclude that starting treatment will probably further reduce the already slightly compromised HRQoL during the watch and wait phase—at least temporarily. That applies to the relatively mild agent chlorambucil, and that decrease might be even bigger for the more effective, but also more intensive therapies that are (coming) available. The expected impact of starting treatment on HRQoL should therefore be considered in the decision whether to start treatment or not.

It is remarkable that the HRQoL is already compromised in untreated patients since in general, treatment is started when the patients experience B-symptoms or disease progression. None of the three previous studies that reported the HRQoL in CLL patients in a non-trial setting, reported the scale scores of HRQoL in untreated patients. We are therefore not able to compare our results in untreated patients with other studies.

When looking at the total group of CLL patients, our results compare very well with those of Holzner et al. [16], who found a lower HRQoL in CLL patients compared with the age- and gender-matched healthy population on 8 of the 15 items/scales of the EORTC QLQ-C30 at baseline. We came to the same conclusion, but we found more statistically significant differences (10 of the 15 items/scales) compared with the general population. However, our patient scores on the EORTC QLQ-C30 were better than those reported by Holzner et al. [16]. This is probably due to the lower age of the patients in our study, and the earlier disease stage at diagnosis.

A recent article by Pashos et al. [17] reported the baseline results of the HRQoL study using the Brief Fatigue Inventory, EQ-5D, and Functional Assessment of Cancer Therapy-Leukemia. Our results on the EQ-5D_5_ are comparable to those reported by Pashos et al. [17].

In the study by Shanafelt et al. [15], CLL patients scored only worse than the general population on the emotional scale of the FACT-G questionnaire. Just like the results of the study by Shanafelt et al., we found a significant difference from the norm score on emotional functioning for the total group of CLL patients, but in contrary to their study, we found many other differences as well.

Fatigue is one of the most frequently reported symptoms among patients with CLL. Our study showed that even untreated patients report significantly more symptoms of fatigue than the general population, and during or after treatment the symptoms were worse. It is a common symptom even many years after diagnosis. More attention should be given to this symptom during and after treatment, but also during the watch and wait phase. Interventions may help to reduce fatigue, but since the precise underlying pathophysiology is largely unknown [31], further studies are necessary.

### Limitations of the study

Since new treatments tend to prolong the overall survival of CLL patients [32], the quality of life during and after treatment becomes more important. Although our study provides insight into the problems that patients with CLL are likely to have, the relatively small number of patients did not allow for comparisons between therapies. This would be very informative for clinicians, but to enable these comparisons in a real-world setting, many patients need to be enrolled, given the high number of available treatments. Due to a low incidence rate of CLL, this would require a long inclusion period or an international approach. Changes in management of CLL over time make it difficult to interpret results of a study with a long inclusion period, and an international study also carries difficulties to the interpretation of the results. Fortunately, the HRQoL results of clinical trials can provide important information on this issue.

A second limitation of our study was that due to the observational character of the study, we were dependent on the health practitioners involved in the study for the timely administration of questionnaires, specifically the questionnaire at the start of a new treatment. Despite our efforts to remind them, they forgot to hand over the questionnaire to the patients before the start of the treatment in the majority of the patients who started a new treatment during our study period. We did not have sufficient information about the HRQoL at the start of treatment to compare the HRQoL before and after treatment.

Another limitation is the uncertainty around the utility scores of the EQ-5D_5_ instrument. To decrease this uncertainty, we also showed the mean utility over the study period using two other methods to generate utility values. The first additional method using a predictive model has been developed in multiple myeloma and validated in non-Hodgkin lymphoma patients. The predicted values appeared to follow a similar pattern to the observed EQ-5D values [33]. The second additional method used the “crosswalk” obtained from an international study of the EuroQol group that administered both the three-level and five-level versions of the EQ-5D (see their website: www.euroqol.org).

The mean utility score of the midpoint estimation and the two additional methods for the total CLL group—based on only those questionnaires without missing values necessary to derive all three estimations—give the following utility scores: 0.854, 0.847, and 0.844. Since these three methods give quite similar results, we can conclude that our calculation is quite reliable.

Since WHO performance status and the presence of comorbidities influence HRQoL, they are potential confounders in our study. We were not able to correct for these potential confounders due to the heterogeneity in treatment patterns resulting in too small patient groups to apply for example propensity score matching. Patients with measurements during the watch and wait phase and those with measurements during treatment with chlorambucil did, however, not differ statistically in WHO performance status and the presence of comorbidities.

### Generalisability

The patient characteristics in our study seem to be reasonably representative for the entire Dutch CLL population since the distribution of gender and the average age at diagnosis agree reasonably well with those of the national registration of CLL and indolent lymphomas (63 vs. 56 % males and 63 vs. 66 years of age) [34]. The slightly lower mean age at diagnosis may be caused by the tendency of haematologists not to bother older patients with the study, or the higher refusal rate to participate by the older patients. The distribution of the disease stages, however, also corresponds with the published distribution in The Netherlands: Binet stage A: 71 versus 60 %, Binet stage B: 16 versus 30 %, and Binet stage C: 11 versus 10 % [35].

In contrast to most RCTs, we also included patients with severe co-morbidity. Co-morbidity (severe heart failure, severe pulmonary disease, severe neurologic disease, severe metabolic disease, inadequate liver function, inadequate renal function, or other co-morbidity) was present in 28 % of the patients. RCTs which aim to study the efficacy of treatments and their influence on HRQoL, often exclude these patients. The outcome of treatments in daily practice could therefore differ from the results found in the RCT. We showed that HRQoL is indeed negatively influenced by having comorbidities and the WHO stage at diagnosis. In our study, the patient group “chlorambucil only” had the highest percentage of patients with co-morbidity. This may explain the relatively worse HRQoL of the patients in this group compared with the patients receiving other treatments.

The percentage of patients with comorbidities was even higher in the group with non-participants. They were also significantly older at diagnosis than participants. This might be related to their choice not to participate in the quality of life study. The percentage of patients willing to participate in the HRQoL study was, however, very high (90 %) so that we do not expect that inclusion of these patients would significantly affect the results.

Since the group of patients with co-morbidity is growing steadily due to an ageing population and an improved overall survival, future research should also focus on the effectiveness of treatments in these patients and the effect of treatments on their HRQoL.

## Conclusion

We concluded that CLL has a profound impact on HRQoL. The HRQoL in CLL patients is compromised when compared with age- and gender-matched norm scores of the general population. Patients with CLL differ most from the general population with regard to the level of role functioning, symptoms of fatigue, concerns about future health, and lacking energy. For patients in the watch and wait phase, the impact of their disease was limited, but larger than generally assumed. In particular with regard to symptoms of sleeping problems and fatigue, more attention should be given to these patients. Once treatment was indicated, HRQoL became considerably compromised. This applied to all treatments, including chlorambucil, which is considered to be a mild treatment. The impact of starting a treatment on the HRQoL should therefore be weighted in the decision whether to start therapy, especially since more effective, but also more intensive therapies are becoming available.

## Electronic supplementary material

Supplementary material 1 (DOCX 13 kb)
