# Supplementary material for: Quality of life of patients with chronic lymphocytic leukaemia in the Netherlands: results of a longitudinal multicentre study
Source: Qual Life Res. 2015 Jul 24;24(12):2895–906. doi: 10.1007/s11136-015-1039-y (PMC4615661; doi:10.1007/s11136-015-1039-y)
Supplement: Supplementary file 1 — Supplementary material 1 (DOCX 13 kb) [file 11136_2015_1039_MOESM1_ESM.docx]

Supplemental Table 1. Utility and VAS scores per category of the included patient characteristics

|  | During Watch and wait phase (N=71) | | During treatment with Chlorambucil (N=42) | | Total group of patients (N=144) | |
| --- | --- | --- | --- | --- | --- | --- |
|  | Utility (SD) | VAS (SD) | Utility (SD) | VAS (SD) | Utility (SD) | VAS (SD) |
| **Total score** | 0.88 (0.13) | 77.37 (12.4) | 0.81 (19.3) | 69.1 (14.5) | 0.85 (0.14) | 73.5 (12.9) |
|  |  |  |  |  |  |  |
| **Gender (p-value)** | **p=0.025** | p=0.132 | p=0.595 | p=0.843 | p=0.068 | p=0.102 |
| Male | 0.91 (0.12) | 79.26 (12.1) | 0.79 (0.23) | 69.5 (16.5) | 0.88 (0.13) | 75.1 (12.9) |
| Female | 0.84 (0.14) | 74.77 (12.4) | 0.82 (0.13) | 68.6 (12.3) | 0.82 (0.14) | 70.9 (12.7) |
|  |  |  |  |  |  |  |
| **Ageclass (p-value)** | **p=0.046** | p=0.290 | p=0.242 | p=0.836 | p=0.721 | p=0.888 |
| <59 | 0.91 | 78.76 (12.6) | 0.83 (0.15) | 71.2 (10.0) | 0.88 (0.10) | 74.1 (12.2) |
| 60-69 | 0.91 (0.10) | 79.66 (11.1) | 0.78 (0.10) | 67.5 (12.1) | 0.86 (0.14) | 73.7 (13.5) |
| >69 | 0.81 | 74.22 (13.0) | 0.79 (0.29) | 67.6 (20.2) | 0.83 (0.17) | 72.7 (13.4) |
|  |  |  |  |  |  |  |
| **WHO status at diagnosis (p-value)** | **p=0.021** | **p=0.004** | p=0.730 | p=0.826 | **p=0.041** | p=0.146 |
| WHO 0 | 0.90 (0.12) | 79.22 (11.4) | 0.81 (0.20) | 69.8 (15.8) | 0.87 (0.13) | 74.8 (12.8) |
| WHO 1 | 0.80 (0.17) | 68.24 (13.5) | 0.78 (0.17) | 68.4 (16.4) | 0.81 (0.15) | 70.6 (11.9) |
| WHO not known | - | - | 0.73 (0.24)* | 59.5 (13.4)* | 0.68 (0.19) | 56.2 (13.7) |
|  |  |  |  |  |  |  |
| **Binet stage at diagnosis (p-value)** | p=0.259 | p=0.742 | p=0.795 | p=0.789 | p=0.375 | p=0.265 |
| Stage A | 0.88 (0.13) | 77.58 (12.5) | 0.79 (0.23) | 67.2 (17.6) | 0.87 (0.14) | 75.1 (13.2) |
| Stage A pr. | - | - | 0.90 (n.a.)* | 75.0 (n.a.)* | 0.87 (0.06) | 70.1 (15.1) |
| Stage B | 1.00 (0.00)* | 71.20 (10.4)* | 0.81 (0.15) | 72.0 (11.3) | 0.82 (0.15) | 68.1 (11.9) |
| Stage C | 0.86 (0.20)* | 76.23 (15.0)* | 0.84 (0.06) | 69.5 (2.5) | 0.85 (0.10) | 72.4 (10.7) |
|  |  |  |  |  |  |  |
| **B-symptoms at diagnosis (p-value)** | p=0.551 | p=0.543 | p=0.302 | p=0.333 | p=0.447 | p=0.276 |
| No | 0.88 (0.13) | 77.18 (12.2) | 0.79 (0.21) | 68.1 (15.2) | 0.86 (0.14) | 73.9 (12.8) |
| Yes | 0.93 (0.11) | 81.67 (18.6) | 0.87 (0.08) | 74.0 (9.7) | 0.84 (0.12) | 71.0 (13.5) |
|  |  |  |  |  |  |  |

* Less than 3 patients within the subgroup. P-values indicate statistical significance between the categories of the patient characteristics (e.g. between age classes).
